# Atypical Human Infections by Animal Trypanosomes

**DOI:** 10.1371/journal.pntd.0002256

**Published:** 2013-09-12

**Authors:** Philippe Truc, Philippe Büscher, Gérard Cuny, Mary Isabel Gonzatti, Jean Jannin, Prashant Joshi, Prayag Juyal, Zhao-Rong Lun, Raffaele Mattioli, Etienne Pays, Pere P. Simarro, Marta Maria Geraldes Teixeira, Louis Touratier, Philippe Vincendeau, Marc Desquesnes

**Affiliations:** 1 Institut de Recherche pour le Développement (IRD), UMR InterTryp 177 IRD/CIRAD, Campus International de Baillarguet, Montpellier, France; 2 Institute of Tropical Medicine, Antwerp, Belgium; 3 Universidad Simón Bolívar, Departamento de Biología Celular, Caracas, Venezuela; 4 World Health Organization, Innovative and Intensified Disease Management, Neglected Tropical Diseases Control, Geneva, Switzerland; 5 Indira Gandhi Government Medical College, Nagpur, India; 6 Guru Angad University, Ludhiana, India; 7 Zhongshan School of Medicine, Sun Yat-Sen University, Guangzhou, The People's Republic of China; 8 Food and Agricultural Organization of the United Nations, Rome, Italy; 9 Université Libre de Bruxelles, Laboratory of Molecular Parasitology, Gosselies, Belgium; 10 University of São Paulo, São Paulo, São Paulo, Brazil; 11 OIE ad hoc Group on Non Tsetse Transmitted Animal Trypanosomoses, Diagnosis, World Organization for Animal Health, Paris, France; 12 Université de Bordeaux 2, UMR InterTryp 177 IRD/CIRAD, Laboratory of Biology and Parasitology, Bordeaux, France; 13 Centre de Coopération Internationale en Recherche Agronomique pour le Développement (CIRAD), UMR InterTryp 177 IRD/CIRAD, Kasetsart University, Bangkok, Thailand; Foundation for Innovative New Diagnostics (FIND), Switzerland

## Abstract

The two classical forms of human trypanosomoses are sleeping sickness due to *Trypanosoma brucei gambiense* or *T. brucei rhodesiense*, and Chagas disease due to *T. cruzi*. However, a number of atypical human infections caused by other *T.* species (or sub-species) have been reported, namely due to *T. brucei brucei*, *T. vivax*, *T. congolense*, *T. evansi*, *T. lewisi*, and *T. lewisi*-like. These cases are reviewed here. Some infections were transient in nature, while others required treatments that were successful in most cases, although two cases were fatal. A recent case of infection due to *T. evansi* was related to a lack of apolipoprotein L-I, but *T. lewisi* infections were not related to immunosuppression or specific human genetic profiles. Out of 19 patients, eight were confirmed between 1974 and 2010, thanks to improved molecular techniques. However, the number of cases of atypical human trypanosomoses might be underestimated. Thus, improvement, evaluation of new diagnostic tests, and field investigations are required for detection and confirmation of these atypical cases.

**Key Learning Points**

- The classical human trypanosomoses are human African trypanosomosis (HAT) or sleeping sickness, and Chagas disease, the Latin American human trypanosomosis.
- Atypical human infections caused by *Trypanosoma* species that normally are restricted to animals have been reported. These cases of atypical human trypanosomoses (a-HT) are mostly transient, but some require treatment and can be fatal.
- Only a few cases of a-HT have been fully confirmed, especially in Asia, leading to the hypothesis that the actual prevalence is probably underestimated.
- The detection of a case of a-HT should be based on observation of the parasite by direct microscopy. Evaluating/improving the diagnoses through serological and PCR assays would help in detecting and identifying atypical trypanosomosis infections in humans. These laboratory research and field activities are needed to evaluate the actual occurrence of atypical cases.

**Top Five Papers**

1. Verma A, Manchanda S, Kumar N, Sharma A, Goel M, et al. (2011) *Trypanosoma lewisi* or *Trypanosoma lewisi*-like infection in a 37-day-old infant. Am J Trop Med Hyg 85: 221–224.
2. Deborggraeve S, Koffi M, Jamonneau V, Bonsu FA, Queyson R, et al. (2008) Molecular analysis of archived blood slides reveals an atypical human *Trypanosoma* infection. Diagn Microbiol Infect Dis 61: 428–433.
3. Vanhollebeke B, Truc P, Poelvoorde P, Pays A, Joshi PP, et al. (2006) Human *Trypanosoma evansi* infection linked to a lack of apolipoprotein L-I. N Engl J Med 355: 2752–2756.
4. Joshi PP, Shegokar V, Powar S, Herder S, Katti R, et al. (2005) Human trypanosomiasis caused by *Trypanosoma evansi* in India: the first case report. Am J Trop Med Hyg 73: 491–495.
5. Howie S, Guy M, Fleming L, Bailey W, Noyes H, et al. (2006) A Gambian infant with fever and an unexpected blood film. PLoS Med 3: e355. doi:10.1371/journal.pmed.0030355.

## Introduction

Trypanosomes are protozoan parasites found worldwide, infecting humans, domestic and wild animals, most often transmitted by blood-sucking insects. The typical pathogenic human trypanosomoses are sleeping sickness, or human African trypanosomosis (HAT) [1], and the Latin American Chagas disease [2]. HAT is a fatal disease occurring in sub-Saharan Africa and transmitted by tsetse flies, caused by two subspecies of trypanosomes: *T. brucei gambiense* (the chronic form) or *T. b. rhodesiense* (the acute form) [1], which is derived from the animal parasite *T. b. brucei* that has acquired the ability to infect humans [1]. Chagas disease, caused by *T. cruzi*, is transmitted by triatomine bugs but also orally [3], congenitally, and via blood transfusion or organ transplantation [2]. The disease is endemic in Latin America and in most cases is chronic and asymptomatic [2]. In addition to these species, *T. rangeli* is also a human infective species, although considered nonpathogenic [1].

In contrast to these species or sub-species, most trypanosomes were thought to be infective only to animals, such as *T. b. brucei*, *T. congolense*, and *T. vivax*, the agents of the complex animal trypanosomosis called “nagana” in Africa. *T. evansi* is responsible for a widely distributed disease called “surra” in domestic and wild animals found in Asia, Africa, South America, and even Europe [4]. *T. lewisi* is a worldwide non-pathogenic parasite of rats transmitted by fleas [1].

Humans possess an innate protection against most *Trypanosoma* species [5]. However, 19 cases of atypical human trypanosomoses (a-HT) caused by *T. b. brucei* [1], [6]–[8], *T. vivax* [1], *T. congolense* [9], *T. evansi* [10]–[13] and *T. lewisi* [14]–[21], which were all considered non-infective to humans, have been reported. In recent years, *T. evansi* and *T. lewisi* have emerged as potentially pathogenic for humans. While some of these cases reviewed herein were transient, six required trypanocidal treatments that were mostly successful, although two patients died [11], [21]. Out of 15 humans cases recorded between 1974 and 2010, nine have been reported since 2003. Some cases were identified by microscopic observation of trypanosomes only and others using molecular tools, as described hereafter.

With the improvement of diagnostic techniques, in particular molecular assays, it is now easier to identify *Trypanosoma* species and, hence, investigate a-HT. Furthermore, the lack of awareness and sometimes difficult access to health care services reinforce the hypothesis that the number of cases of a-HT might be underestimated. Therefore, it was decided to review these cases of a-HT, leading to an international collaboration to further evaluate their actual occurrence.

## Methods

References for this article were identified through PubMed searches for articles published from 1978 to 2011 using the terms “*Trypanosoma*”, “human”, “atypical”, “*lewisi*”, “*evansi*”, “*congolense*”, and “*Herpetosoma*”. Relevant books and articles published between 1933 and 2011 were selected through searches in the authors' personal files.

### Accession Numbers

Accession numbers on Genbank, for the ITS1 (ribosomal DNA) of *T. lewisi* mentioned in the manuscript are: HQ437158 (*T. lewisi* specific primers), GU252222.1 (human infant case in Thailand), GU252216.1–GU252221.1, DQ345394.1, FJ011094.1, EU861192.1, EU599639.1, FJ011095.1. Human serum associated protein (SRA) CAD90580.1, GI34368410. ApoL1 gene ID 8542, ApoL1 protein AAI4340.1. RoTat 1.2 variable surface glycoprotein (VSG) CAI34904.

### Identification of Atypical Human Cases of Trypanosomoses by Microscopic Observation

**Figure 1:**
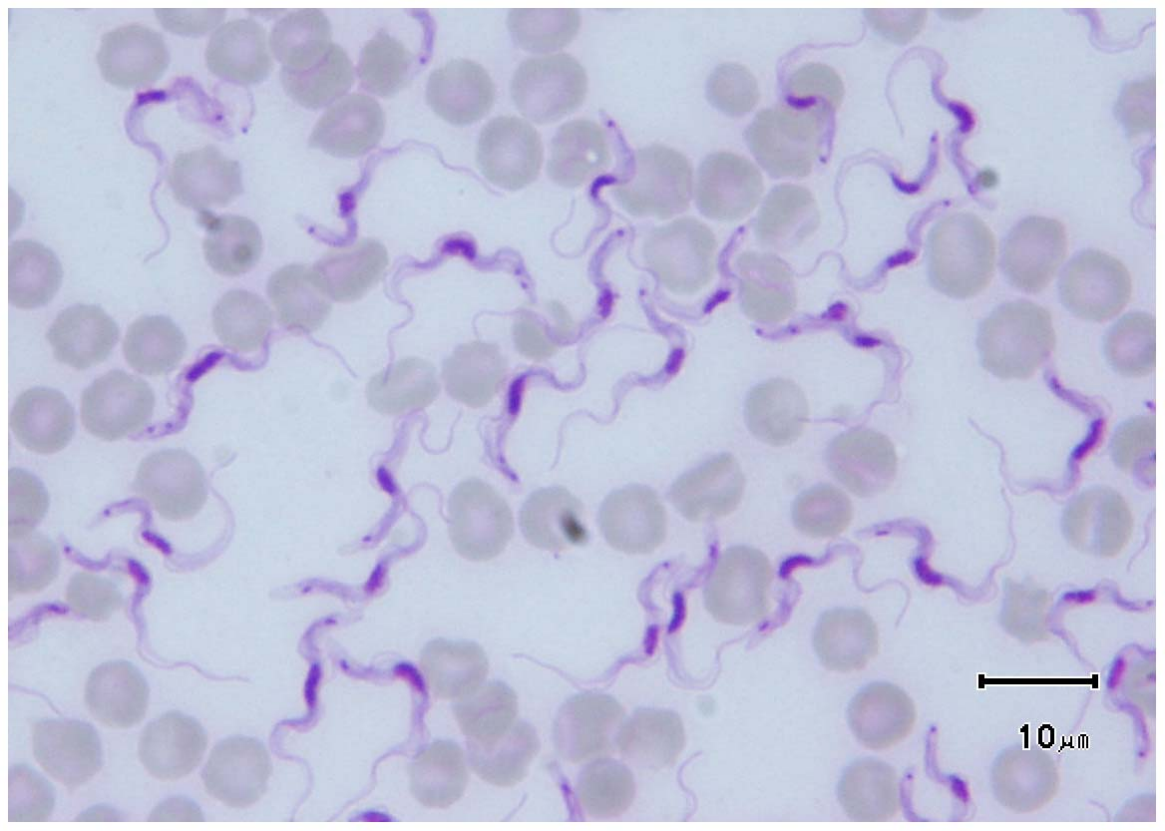
*T.* *evansi* on a Giemsa-stained blood smear from an experimentally infected rat (credit, Marc Desquesnes). Size of the parasite (15–34 µm), small (diameter 0.6–0.7 µm) and subterminal kinetoplast, sharp posterior end, central nucleus, large undulating membrane, and free flagellum are the most striking features of the slender forms of the sub-genus *Trypanozoon*.

**Figure 2:**
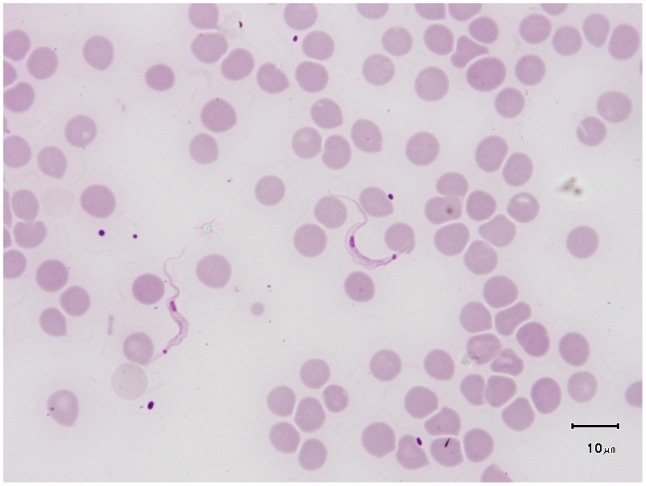
*T.* *lewisi* (adult form) on a Giemsa-stained blood smear from an experimentally infected rat (credit, Marc Desquesnes). Size of the parasite (21–36 µm), kinetoplast rode shaped, large (1×0.7 µm) and subterminal, very sharp posterior end, posterior nucleus, large undulating membrane, “C shape” of the parasite, and free flagellum are the most striking features of the adult forms of the sub-genus *Herpetosoma*, to which belong *T. lewisi*-like parasites.

The 19 atypical human cases of trypanosomoses detected by microscopy reported so far are presented in Table 1, and morphology of *T. evansi* and *T. lewisi* is shown in Figure 1 and Figure 2, respectively (as observed in infected rodents). Among them, 11 cases were identified by morphological analysis of trypanosomes only. Unfortunately, most of these cases were poorly described, such as a case of human infection by *T. vivax* (Table 1, patient number 1) from Ghana in 1917 [1]. In 1930, Mesnil reported an accidental *T. b. brucei* infection of a technician (patient number 2) using a syringe containing infected blood [1]. In 1947, out of seven volunteers infected with *T. b. brucei* by bites of infected tsetse flies, one (patient number 3) acquired a transient infection for 3 weeks [6]. In 1988, the blood incubation infectivity test [22] of an autochthonous sleeping sickness case in Western Ethiopia indicated that the patient was infected with *T. b. brucei* (patient number 4). This patient was cured but no information was given on clinical signs and treatment [7].

**Table 1:** The 19 cases of atypical human trypanosomoses.

Patient Number	Location	Trypanosome Species/Sub-species	Date	Parasite Identification Method^a^	Fever	Treatment	Outcome	Reference Number
1	Ghana	*T. vivax*	1917	Morphology	ND	ND	ND	[1]
2	Pasteur Institute	*T. b. brucei*	1930^b^	Morphology	ND	ND	ND	[1]
3	Congo	*T. b. brucei*	1947^c^	Morphology	Present	None	Self-cure	[6]
4	Ethiopia	*T. b. brucei*	1987	Morphology BIIT	ND	ND	Cure	[7]
5	Ghana	*T. b. brucei*	2003	PCR	Present	None	Self-cure	[8]
6	Côte d'Ivoire	*T. congolense*	1998	PCR	Present	Pentamidine	Cure	[9]
7	India	*T. evansi*	1977^b^	Morphology	Present	Atoxyl	Cure	[10]
8	Sri Lanka	*T. evansi*	1999	Morphology	Present	None	Self-Cure	[11]
9	India, Seoni	*T. evansi*	2004	PCR	Present	Suramin	Cure	[13]
10	India, Kolkata	*T. evansi*	2005	Morphology	Present	None	Death	[11]
11	Egypt	*T. evansi*	2010	Morphology	Present	ND	Cure	[12]
12	Malaysia	*T. lewisi*	1933	Morphology	Present	None	Self-cure	[14]
13	India, Parsda	*T. lewisi*	1974	Morphology	Present	None	Self-cure	[15]
14	India, Parsda	*T. lewisi*	1974	Morphology	Present	None	Self-cure	[15]
15	The Gambia	*T. lewisi*-like	2003	PCR/S	Present	Melarsoprol	Cure	[18]
16	Thailand	*T. lewisi*-like	2003	PCR/S	Present	Antibiotic	Cure	[19]
17	India, Mumbai	*T. lewisi*	2006	Morphology	Present	None	Self-cure	[16], [17]
18	India, Pune	*T. lewisi*	2007	PCR	Present	Suramine	Death	[21]
19	India, Bagpat	*T. lewisi*	2010	PCR/S	Present	Pentamidine	Cure	[20]

a Microscopy of blood smear and morphology of trypanosomes.

b Accidental inoculation with a syringe.

c Volunteer exposed to tsetse bites.

BIIT, blood incubation infectivity test; ND, no data; PCR, PCR molecular assays; PCR/S, PCR molecular assays followed by sequencing of amplicons.

*T. lewisi* was found in the blood of three patients suffering from short febrile episodes in Malaysia (patient number 12 in 1933) and India (patients number 13 and 14 in 1974) [14], [15]. Infection was only transient and patients recovered without treatment. In 2006, trypanosomes were detected in blood of a 7-week-old baby (patient number 17) living in Mumbai (India). She was suffering from fever and presented hepatosplenomegaly. Morphological examination of trypanosomes indicated *T. lewisi* or *T. lewisi*-like. The patient self-cured in 15 days [16], [17].

**Figure 3:**
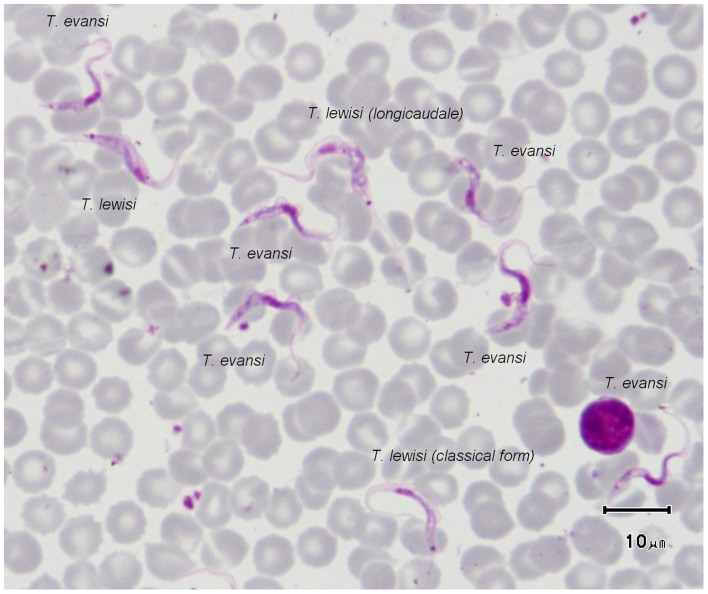
*T.* *lewisi* and *T. evansi* on a Giemsa-stained blood smear from a rat submitted to a mixed experimental infection (credit, Marc Desquesnes). Smeared and stained in the same conditions, the parasites exhibit here their striking characteristics: the size of the kinetoplast (large in *T. lewisi* and small in *T. evansi*) is the most reliable criteria to distinguish these sub-genera; however, other characteristics can be observed, such as: the size of the parasite (*T. lewisi*>*T. evansi*), the nucleus position (central for *T evansi*, posterior for *T. lewisi*), the undulating membrane development (exhibiting regular waves in *T. evansi* and irregular ones in *T. lewisi*). Finally, an unusual feature can be observed on one specimen of *T. lewisi* with a very long posterior extremity, this morphotype use to be called “*longicaudale*” [1].

In 1977, an accidental infection by a syringe containing *T. evansi*-infected blood was reported in India. The patient (patient number 7) was treated with Atoxyl (an arsenic derivative used in HAT treatment before the currently used melarsoprol drug) [10]. In 1999, a human suspected of being infected with *T. evansi* was reported in Sri Lanka (patient number 8). He suffered from headache and episodes of hyperthermia concomitant to high levels of parasitaemia. Again, the case was not thoroughly documented [11]. In 2005, another case of *T. evansi* was reported in West Bengal State, India. The patient died 2 days after admission to a Kolkata hospital (patient number 10), but it is only suspected that death was caused by the related trypanosome infection [11]. In 2010, in Egypt, a cattle farmer was infected with *T. evansi* and cured; however, this case (patient number 11) remains doubtful because no attempts were made to ascertain the parasite species using molecular techniques and no clinical details or the drug used for treatment were given [12]. Reliable criteria such as the size and the position of the kinetoplast, the location of the nucleus, the development of the undulating membrane, the shape of the posterior end of the parasite, the existence of free flagellum, etc. (Figure 3), may allow sub-genus identification based on the morphological features of the parasites. However, the species identification is not possible, and, the parasitemia is sometime too low for such observations to be conclusive. Consequently, molecular identification of trypanosomes is most often necessary.

### Molecular Assays to Confirm Atypical Human Cases of Trypanosomoses

In 1998 a mixed infection with *T. brucei* spp. and *T. congolense* was detected in the blood of a patient (patient number 6) in Côte d'Ivoire. The latter trypanosome species was confirmed by PCR assays whereas *T. brucei* was only identified at the species level; therefore *T. b. gambiense* cannot be ruled out. The patient was successfully treated using pentamidine [9]. In 2003 in Ghana, a patient suspected of having HAT (patient number 5) who recovered without treatment was later confirmed to be infected with *T. b. brucei* by molecular analysis of archived blood slides [8]. The number of potentially similar cases on the African continent remains difficult to evaluate. Conventional diagnosis of HAT is based on microscopic detection of trypanosomes in blood, lymphatic fluid, and/or cerebrospinal fluid (CSF). This technique used in medical surveys does not allow discrimination between *T. b. brucei*, *T. b. gambiense*, and *T. b. rhodesiense*, since they are morphologically indistinguishable [1].

In 2004, the first molecularly confirmed case of a-HT caused by *T. evansi* was described in Seoni, India, in a farmer (patient number 9) showing a fluctuating trypanosome parasitaemia associated with febrile episodes over the course of 5 months [13]. The same signs were observed in a Sri Lankan patient in 1999 (patient number 8). Microscopical examination of blood smears showed high numbers of parasites confirmed as *T. evansi* by molecular techniques [23]. Additional examinations did not show central nervous system invasion by the parasites and the patient was successfully treated with suramin [24].

In 2003, trypanosomes were detected in blood and CSF of a Gambian baby presenting a severe clinical status with general oedema although no neurological abnormalities were found (patient number 15). PCR assays with primers flanking trypanosome ribosomal DNA internal transcribed spacer 1 (ITS1) resulted in amplicon of ∼623 bp that corresponded to the expected size for *T. lewisi* and allied species (*T. lewisi*-like) [19], which are closely related species of the subgenus *Herpetosoma*; the amplified ITS sequence differed by just one nucleotide from *T. lewisi* [25]. The patient was successfully treated with melarsoprol [18]. Two other babies were reportedly infected with *T. lewisi* or *T. lewisi*-like. In 2003 in Thailand, trypanosomes were observed in blood from a 45-day-old infant (patient number 16) displaying fever, anaemia, cough, and anorexia. PCR (ITS1) confirmed the *T. lewisi*-like infection. The patient was treated with gentamicin [19]. In Bagpat India, 2010, a 37-day-old infant (patient number 19) with fever, anorexia, and lethargy showed *T. lewisi* on blood smears. Analysis of the DNA sequence (ITS1) amplified by PCR with this infant's blood confirmed the species identification. The patient was treated using pentamidine for 10 days, and on the seventh day peripheral blood smear did not show any parasites [20]. In 2007, in Pune, India, an adult (patient number 18) was infected by *T. lewisi* detected by microscopy of blood smears and confirmed by PCR assay (not detailed in the publication). However, treatment with suramin had to be interrupted because of renal complications leading to the patient's death [21]. This indicates prudence is required when managing a-HT. Toxicity of the drugs and parasite-related pathogenicity must be assessed.

### Immunity of Humans to African Animal Trypanosomes

The natural immunity of humans to the livestock pathogen *T. b. brucei*, but not to the morphologically indistinguishable human pathogens *T. b. gambiense* and *T. b. rhodesiense*, is due to the selective killing of the parasite by normal human serum (NHS). While the mechanism is still not known in the case of *T. b. gambiense*, a truncated protein named serum resistance-associated (SRA) appears to be the dominant factor responsible for resistance of *T. b. rhodesiense* to NHS [26]. Human innate immunity against African animal trypanosomes stems from the trypanolytic activity of the human-specific serum protein called apolipoprotein L-I (apoL-I), which is partially associated with high-density-lipoprotein (HDL) [5], [27]–[29].

However, under certain circumstances, it would appear that *T. congolense*, *T. vivax*, and *T. evansi* can be resistant to human plasma [30]–[33]. The serum of an Indian patient (Table 1, number 9) infected with *T. evansi* presented a lack of trypanolytic activity due to frameshift mutations in both *apoL*-I alleles. Therefore, the lack of efficient apoL-I can explain this human *T. evansi* infection [34]. More isolates of *T. evansi* from various regions should be tested to check their sensitivity to NHS, and prevalence of *apoL-I* mutations should be investigated. Similarly, the trypanolytic activity of NHS from various geographical origins could be evaluated against a reference strain of *T. evansi* and the prevalence of the *apoL*-I deficit should be investigated in several human populations, to evaluate the potential risk of *T. evansi* infection in humans.

### Immunity to *T. lewisi* and *T. lewisi*-Like Parasites

In contrast to *T. b. brucei*, little is known about the innate immune response that prevents the establishment of human infection by other trypanosome species. *T. lewisi* and *T. lewisi*-like species are in general highly host-restricted to rodents and lagomorphs. *T. lewisi* is primarily a parasite of *Rattus* spp. but little is known about the mechanisms involved in the highly host-species restriction of *T. lewisi* and related species and, consequently, why other animal species including primates are not naturally infected under normal conditions.

During the course of infection in the rat, *T. lewisi* produces two antigenic variants: the first represents the initial reproducing population and the second, the non-reproducing population. The reproductive forms are inhibited by ablastin [35] and the late population by antibody dependent cytotoxicity. The capacity to evade trypanocidal and ablastic antibodies and complement is crucial in the establishment of the infection. In rats infected with *T. lewisi* the parasitaemia normally resolves within 30 days. Thereafter, the rodents become immune to re-infection and complement does not appear to play a major role in this process [36], [37]. However, the inability of *T. lewisi* to infect a range of mammalian species appears to be due to the activation of complement through the alternative pathway, agglutinins, and opsonins [36]. *T. lewisi* infection of non-natural hosts, including non-human and human primates, apparently, lasts a very short time compared to infections in natural hosts [36], [38]. Infection with *T. lewisi* was also transient in some patients from Malaysia and India who recovered without treatment [14]–[17]. It seems that only immature or depressed immune systems could render this species an opportunistic parasite of primates [37]. However, human resistance mechanisms against infection by this species have not been investigated yet.

### Investigations in Rodents

Following the detection of *T. lewisi* in a sick Thai infant (Table 1, patient number 16), *T. lewisi* infection in rodents was investigated to identify possible sources of human cases in Thailand. Blood samples from 276 rodents were tested with PCR (ITS1), and the trypanosome species identified by ITS1 sequence analysis. *T. lewisi* was detected in *Rattus* spp. (14.3%) and *Bandicota* spp. (18.0%). The ITS1 sequence from one sample from *R. tanezumi* showed 96.4% similarity compared to the sequence amplified from the blood of the *T. lewisi*-infected Thai infant [19], [38]. Habitats where rodents were collected suggested that the degree of anthropization may influence the transmission of *T. lewisi* [39]. The hypothesis that *T. lewisi* can be transmitted from rats to non-human primates by rodent fleas arose from the finding of *T. lewisi* infection in captive monkeys infected by fleas, and living in poor conditions in rat-infested cages [37]. This hypothesis is corroborated by the fact that humans infected with *T. lewisi* also lived in poor dwellings certainly infested by domestic rats [19].

### Improving Diagnostic Methods for a-HT

Several techniques such as IFAT, ELISA [40], [41], and PCR-based methods [42] have been developed for the detection of trypanosomes in animals, especially for *T. evansi*. Serological techniques making use of crude antigens proved to have strong inter-specific cross-reactions [43]. They are therefore not considered as species-specific, although their genus specificity is satisfactory [43] and some improvements are expected for more specific antigens such as VSG Rotat 1.2 *T. evansi* [44]. Few of these techniques have been applied to a-HT.

In 2005, a serological survey was conducted using the Card Agglutination Test for Trypanosomosis/*Trypanosoma evansi* (CATT/*T. evansi*) [45] in the residential area of the patient number 9 (Indian case of *T. evansi* infection in Seoni [13]). Out of 1,806 individuals tested with CATT/*T. evansi*, 81 were positive using serum [46]. No trypanosome was detected in the blood of 60 persons positive to the test at a significant serum dilution (minimum 1∶4). These results may suggest a frequent human exposure to *T. evansi* in the study area and possibly a frequent transmission of parasites to humans leading to transient infections in a “normal” immune population. The specificity of the CATT/*T. evansi* test has not been previously evaluated for human screening and was used for the first time in this study in Asia. Consequently, this and other diagnostic techniques must be evaluated for screening a-HT in human populations, such as ELISA-*T. evansi*, and the immune trypanolysis test with *T. evansi* RoTat 1.2, which is considered to be highly specific for *T. evansi* infection in livestock but cannot detect some strains of *T. evansi* when the RoTat 1.2 VSG gene is missing [47]. So far, species-specific identification of *Trypanosoma* species can only be implemented by PCR with various sets of primers [48], or by sequence analyses of PCR products [37].

More recently, molecular tools were developed for the species-specific identification of *T. lewisi* by PCR or LAMP techniques [38], [49]. These methods are under evaluation in several rodent species and might be useful in humans. Species-specific diagnoses will allow a better assessment of the prevalence of a-HT and a more accurate characterization of cases. They could also be useful to assess the results of potential treatments, with the goal of improving the management of any emerging a-HT infection.

## Conclusion

The number of a-HT cases attributable to primarily animal trypanosomes is possibly underestimated. An international collaboration could help develop tools and strategies to better detect infection, identify the causative species, and manage new cases. The risk and potential impact related to a-HT cannot be evaluated thoroughly at the present time because diagnostic, clinical, and epidemiological data remain insufficient. Further studies are required to address relevant aspects of a-HT: (i) enhance awareness and detection in suspected areas where a-HT could be prevalent; (ii) real-time and detailed reports of a-HT including the clinical history of the patients; (iii) easy, sensitive, and species-specific methods for identification of the trypanosome; (iv) investigations on potential vectors, reservoirs (wild and domestic animals), and infection pathway. Therefore, it is perfectly justified to establish an international network to evaluate whether a-HT could be an emerging and neglected threat to human health.
